# Genome-Wide Association Studies Reveal that Diverse Heading Date Genes Respond to Short and Long Day Lengths between *Indica* and *Japonica* Rice

**DOI:** 10.3389/fpls.2016.01270

**Published:** 2016-08-29

**Authors:** Zhongmin Han, Bo Zhang, Hu Zhao, Mohammed Ayaad, Yongzhong Xing

**Affiliations:** ^1^National Key Laboratory of Crop Genetic Improvement and National Center of Plant Gene Research, Huazhong Agricultural UniversityWuhan, China; ^2^Hubei Collaborative Innovation Center for Grain IndustryJingzhou, China

**Keywords:** heading date, long and short-day conditions, genome-wide association studies, haplotype-level association

## Abstract

Rice is a short-day plant. Short-day length promotes heading, and long-day length suppresses heading. Many studies have evaluated rice heading in field conditions in which some individuals in the population were exposed to various day lengths, including short and long days, prior to a growth phase transition. In this study, we investigated heading date under natural short-day conditions (SD) and long-day conditions (LD) for 100s of accessions and separately conducted genome-wide association studies within *indica* and *japonica* subpopulations. Under LD, three and four quantitative trait loci (QTLs) were identified in *indica* and *japonica* subpopulations, respectively, two of which were less than 80 kb from the known genes *Hd17* and *Ghd7*. But no common QTLs were detected in both subpopulations. Under SD, six QTLs were detected in *indica*, three of which were less than 80 kb from the known heading date genes *Ghd7*, *Ehd1*, and *RCN1*. But no QTLs were detected in *japonica* subpopulation. *qHd3* under SD and *qHd4* under LD were two novel major QTLs, which deserve isolation in the future. Eleven known heading date genes were used to test the power of association mapping at the haplotype level. *Hd17*, *Ghd7*, *Ehd1*, and *RCN1* were again detected at more significant level and three additional genes, *Hd3a*, *OsMADS56*, and *Ghd7.1*, were detected. However, of the detected seven genes, only one gene, *Hd17*, was commonly detected in both subpopulations and two genes, *Ghd7* and *Ghd7.1*, were commonly detected in *indica* subpopulation under both conditions. Moreover, haplotype analysis identified favorable haplotypes of *Ghd7* and *OsMADS56* for breeding design. In conclusion, diverse heading date genes/QTLs between *indica* and *japonica* subpopulations responded to SD and LD, and haplotype-level association mapping was more powerful than SNP-level association in rice.

## Introduction

Rice as a short-day plant is cultivated from latitudes of 55°N in China to 36°S in Chile (Khush, 1997). Rice heading date is an important trait to adapt to various regional environments and is usually related to grain yield. Many isolated QTLs showed varying photoperiod sensitivities between SD and LD. Under SD, heading date is promoted by two independent pathways mediated by *Heading date 1* (*Hd1*) and *Early heading date 1* (*Ehd1).* *Hd1* is a homolog of *CONSTANS* in *Arabidopsis*. *Ehd1* encoding a B-type response regulator has no ortholog in *Arabidopsis*. *Hd1* and *Ehd1* up-regulated the expression of the FT-like (florigen) *Hd3a* genes (Yano et al., 2000; Doi et al., 2004). *Ehd2*, *Ehd3*, and *Ehd4* promote heading by up-regulating *Ehd1* under SD conditions (Matsubara et al., 2008, 2011; Gao et al., 2013). Most day-length-sensitive heading date QTLs, such as *Hd6*, *Ghd7*, *OsMADS56*, *OsMADS50*, *Hd17*, *DTH2*, *Hd16*, and *Ghd7.1*, function specifically under LD (Takahashi et al., 2001; Lee et al., 2004; Xue et al., 2008; Ryu et al., 2009; Matsubara et al., 2012; Hori et al., 2013; Wu et al., 2013; Zhang et al., 2013). Among these QTLs, *OsMADS50*, *Hd17*, and *DTH2* promote heading but the others delay heading under LD. Interestingly, both *Hd1* and *DTH8/Ghd8* delay heading under LD and promote heading under SD (Yano et al., 2000; Wei et al., 2010).

A genome-wide association study (GWAS) combining high-density markers with a diverse germplasm collection provides higher mapping resolution than conventional QTL mapping based on bi-parent derived segregating populations and enables the prediction or identification of causal genes. GWAS has been widely used to identify loci related to various important agronomical traits in rice (Huang et al., 2010, 2012; Zhao et al., 2011). For heading date, Huang et al. (2012) associated three known heading date genes of *OsGI*, *Hd3a*, and *RCN1* with worldwide rice varieties in natural conditions. They were all detected in *indica* subpopulation but *OsGI* was also detected in *japonica* subpopulation. However, the major gene *Ghd7* was not detected. Zhao et al. (2011) conducted experiments in three environments: LD (12–14 h), 12–13 h day-length condition in the field and one very long to very short day-length condition (~18–6 h) in a greenhouse. They identified 10 candidate heading date genes. *Hd1* was the only one repeatedly detected in multiple environments. In both studies, the power of genome-wide association mapping for heading date QTLs is low because few known genes have been identified. The low power of association mapping at the single-nucleotide polymorphism (SNP) level is primarily caused by low resolution in grouping samples into two genotypes. Notably, for adaptive genes in rice such as *Ghd7* and *Ghd7.1*, several alleles/haplotypes exist in nature. Each allele has a different genetic effect and results in a specific geographic distribution (Xue et al., 2008; Zhang et al., 2013). Therefore, association mapping at the haplotype level is expected to be more powerful because the germplasm collection can be classified into several genotypes (as opposed to two genotypes via SNP). Additionally, the heading date data collected from the population grown under mixed LD and SD conditions for GWAS likely leads to deviation because some heading date genes such as *Hd1* have opposing effects on heading between SD and LD, which eliminate their genetic effects and result in failed detection.

*Indica* and *japonica* rice varieties have distinct growing zones and cropping seasons. It is naturally questioned whether different genes control heading date between *indica* and *japonica* subpopulations under LD and SD. To answer the questions, heading date was recorded for a global rice collection under SD and LD in this study, and GWAS for heading date was independently performed in *indica* and *japonica* subpopulations under SD and LD. To check the power of haplotype-level GWAS, 11 known heading date genes were tested. Our results revealed a diverse genetic basis for heading date between *indica* and *japonica* subpopulations under two conditions and a greatly improved power of haplotype-level GWAS as compared to SNP-level GWAS.

## Materials and Methods

### Plant Material, Field Experiments, and Heading Date Record

A diverse worldwide collection of 529 *Oryza sativa* accessions were planted on April 17 in a bird net-equipped field in the experimental farm of Huazhong Agricultural University, Wuhan, China (114° 21′ E, 30° 28′ N) during the summer seasons of 2013 and 2014 and in the winter season, 2012–2013 (on December 5) in Hainan Island, China (110° 01′ E, 18° 30′ N). Field trials were carried out following a randomized complete block design with two replications within each year. The seedlings of each accession were transplanted after 25 days into one row in the field, with a distance of 16.5 cm × 26.4 cm within and between the rows. The heading dates of five plants in the middle of each row were individually recorded. Every 2 days, the heading date was recorded as the days from sowing to the first panicle appearance. The average trait value for each accession across two replicates within each year was used for separate GWAS.

### SNP Database

In total, 529 *O. sativa* landrace and elite accessions were genotyped via sequencing (Chen et al., 2014). The SNP information is available on RiceVarMap^1^, a comprehensive database of rice genomic variations, while the SNP physical locations were cited using the genome of TIGR Rice Loci 6.

### Genome-Wide Association Study

A total of 2,767,159 and 1,857,845 SNPs (minor allele frequency ≥0.05; the number of accessions with minor alleles ≥6) were used for GWAS in *indica* and *japonica* subpopulations, respectively (Chen et al., 2014). Linear mixed models (LMM) were used to make associations by running the Fast-LMM program (Lippert et al., 2011). Population structure was controlled using a kinship matrix constructed with all SNPs. Using a method described by (Li et al., 2012), the effective numbers of independent SNPs were calculated as 571,843 and 245,348 for both *indica* and *japonica* subpopulations, respectively. The suggested *P*-values were specified as 1.8 × 10^-6^ in *indica* and 1.3 × 10^-6^ in *japonica* (Chen et al., 2014). The thresholds were then set at *P* = 8.0 × 10^-7^ in Wuhan and *P* = 3.8 × 10^-7^ in Hainan to identify significant association signals via LMM. To obtain independent association signals, multiple SNPs exceeding the threshold in a 5-Mb sliding window were clustered by r^2^ of linkage disequilibrium ≥0.25, and SNPs with the minimum *P*-value in a cluster were considered lead SNPs. The detailed method was previously described (Chen et al., 2014; Wang et al., 2015).

### Haplotype and Statistical Analyses

SNPs within genes used to define haplotypes for 11 known heading date genes including *Hd17*, *Ghd7*, *Ehd1*, *RCN1*, *Hd1*, *Ghd8*, *Ghd7.1*, *DTH2*, *Hd3a*, *OsMADS50*, and *OsMADS56* were downloaded^2^; the haplotypes were then reconstructed using PHASE software (Stephens et al., 2001) to do imputation for the missing and heterozygous SNPs. The haplotypes with an allele frequency ≥0.01 (5 accessions) were included for association analysis via ANOVA; then, a Duncan test (*Post-hoc* analysis) was conducted to show the difference in heading date between all possible haplotype pairs.

For each condition, the lead SNPs of the associations detected via GWAS were fit into a linear model using an lm function in R software^3^, and the phenotype variations explained by each lead SNP and by all lead SNPs together were estimated.

## Results

### Day Length of the Rice-Growing Season in Hainan and Wuhan

The day length of 13.5 h was set as the boundary between SD and LD (Itoh et al., 2010). Throughout the winter growing season (December–April) in Hainan, the day length ranged from 11.0 to 12.5 h, which is a typical SD. Therefore, all 529 accessions completed their life cycles under SD in Hainan. However, in Wuhan, the summer season day length increased from 13.0 to 14.2 h (April 17–June 22) and then decreased from 14.2 to 12.2 h (June 22–September 21). Therefore, some accessions completed heading under LD; however, some accessions first encountered LD and then experienced SD prior to heading.

Approximately 25 days were required for rice to complete panicle development from the growth phase transition to heading (Vergara et al., 1966; Chang et al., 1969; Liu et al., 2013). In Wuhan, the day length decreased from 13.5 h after August 6, thus the accessions that completed heading prior to August 30 initiated the transition from a vegetative to a reproductive phase under LD. To precisely evaluate the effects of LD conditions on heading date, we removed 26 accessions that experienced mixed LD and SD prior to the phase transition and kept 503 accessions that completed the phase transition under LD (headed prior to August 30) in Wuhan.

### Variation of Heading Date in 503 Diverse Accessions

**FIGURE 1:**
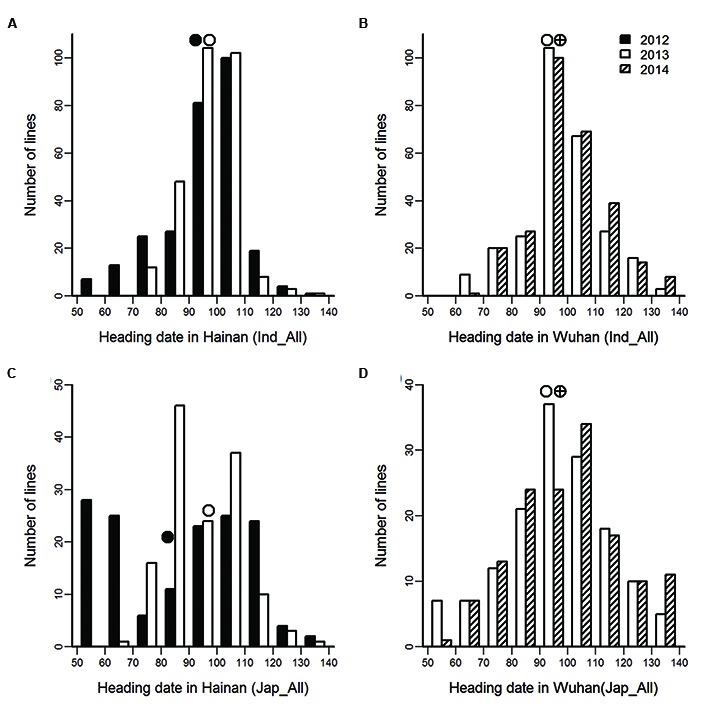
**Performance of heading date in the *indica* (A,B) and *japonica* (C,D) subpopulations.** The circles indicated mean values of heading date.

A structural analysis indicated that the entire collection was divided into several distinct subpopulations such as *indica*, *japonica*, *aus* and mixture (Chen et al., 2014). Heading date exhibited large variation in the 503 accessions, the mean values of heading date in the *indica* subpopulation were equivalent to those in *japonica* rice either in Wuhan or Hainan, with the exception of heading date in 2012 in Hainan (**Figure 1**). The correlation coefficient of heading date between the 2 years was 0.94 in Wuhan and 0.86 in Hainan. The correlation coefficients between different locations were significant but substantially lower than that between years in the same locations (**Table 1**). Two-way ANOVA showed significant genotype and environment effects on heading date (**Table 2**). The genetic factor accounted for the most phenotypic variance (59.5%) and the environmental effect contributed 4.6% of the phenotypic variance, indicating that many accessions showed differing heading dates between the four environments.

**Table 1:** Correlations of heading dates of 503 accessions in different environments.

Environments	HN2013	WH2013	WH2014
HN2012	0.86	0.37	0.31
HN2013		0.44	0.42
WH2013			0.94

**Table 2:** Genotypes and environments analysis of variance for heading date using 503 accessions.

SV	Heading date		
	SSG/SST (%)	*F*	*P*
G	59.5	4.8	<0.0001
E	4.6	63.5	<0.0001

*SV, source of variance; G, genotype; E, environment*.

### QTL Detection Using GWAS in Wuhan (LD)

**FIGURE 2:**
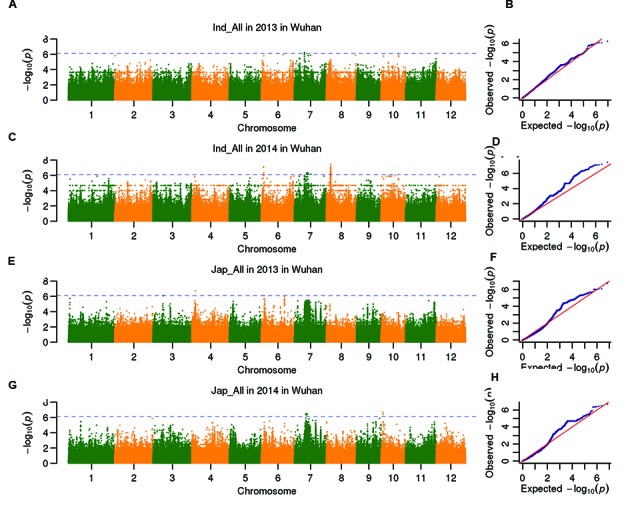
**Genome-wide *P*-values (A,C,E,G) and QQ plots (B,D,F,H) from the LMM model for heading date in *indica* and *japonica* in Wuhan are demonstrated in the four panels.** The horizontal dashed line **(A,C,E,G)** indicates the genome-wide significance threshold (*P* = 8.0 × 10^7^).

The 278 *indica* and 147 *japonica* accessions (after removed 78 of the *aus* or admixed subpopulations) were utilized for GWAS to avoid structure noise. GWAS was separately performed in *indica* and *japonica* subpopulations. A total of seven associations with heading date were detected in Wuhan, three and four QTLs were detected in *indica* and *japonica* subpopulations, respectively (**Figure 2**; **Table 3**). Two QTLs (*qHd6.1* and *qHd7.1*) on chromosomes 6 and 7 were repeatedly detected in *indica* subpopulation and individually explained 18.7–19.7% and 7.1–7.3% of the phenotypic variance, respectively. The lead SNP Sf602312322 for *qHd6.1* was 78.2 kb to *Hd17*, and the lead SNP Sf709177919 for *qHd7.1* located in 25.5 kb from *Ghd7*. In *japonica* subpopulation, *qHd6.2* was repeatedly associated with the heading date in 2 years, however, there are no reported heading date genes nearby *qHd6.2*. *qHd4* was detected only in 2013 but exhibited the largest contribution (26.1%) to the variation in heading date. No common association was detected in either subpopulation. The QTLs detected in *indica* subpopulation cumulatively explained 27.1 and 35.3% of the phenotypic variations in 2013 and 2014, respectively. The QTLs in *japonica* subpopulations explained more variation in the heading date (39.4 and 40.8% in the 2 years, respectively).

**Table 3:** Genome-wide significant association signals for heading date in *indica* and *japonica* subpopulations using the LMM method in Wuhan (long day conditions).

QTL	Lead SNP	Chr.	Linkage disequilibrium interval (bp)	Distance to gene	*Indica* 2013		*Indica* 2014		*Japonica* 2013		*Japonica* 2014	
					P	P.V (%)	P	P.V (%)	P	P.V (%)	P	P.V (%)
*qHd4*	Sf0403512804	4	3145576–3733894						1.8E-7	26.1		
*qHd6.1*	Sf0602312322	6	2311322–2931955	78.2 kb to *Hd17*	1.6E-6	19.7	4.4E-7	18.7				
*qHd6.2*	Sf0602663020	6	2396433–2733565						8.0E-7	13.2	4.4E-6	16.8
*qHd7.1*	Sf0709177919	7	9137593–9196135	-25.5 kb to *Ghd7*	5.7E-7	7.3	1.1E-6	7.1				
*qHd7.2*	Sf0711412802	7	11316108–11898697								3.1E-7	16.6
*qHd8*	Sf0804089477	8	3942483–4245642				3.5E-8	9.5				
*qHd10.1*	Sf1001634949	10	1596335–1634949								2.2E-7	7.4

Total estimation					7.2E-19	27.1	4.0E-25	35.3	3.8E-16	39.4	3.2E-15	40.8

*Linkage disequilibrium interval means the linkage disequilibrium (r^2^ > 0.2) extended genome region surrounding the lead SNP. P is P-value estimated in LMM, P.V (%) is phenotype variance explained by the QTL in percent.*

### QTL Detection via GWAS in Hainan (SD)

**FIGURE 3:**
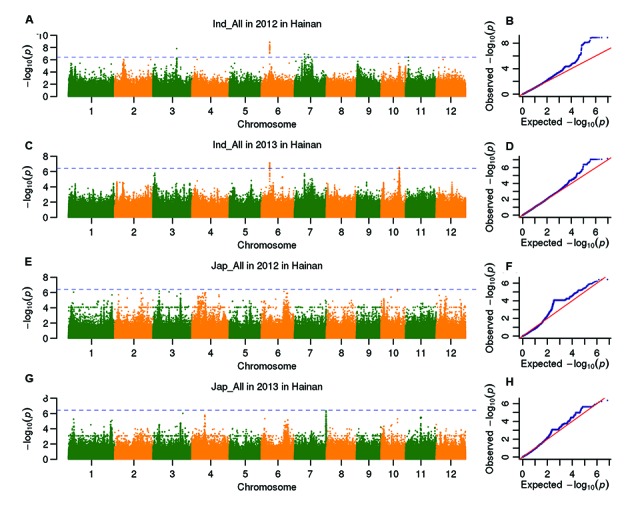
**Genome-wide *P*-values (A,C,E,G) and QQ plots (B,D,F,H) from the LMM model for heading date in *indica* and *japonica* in Hainan are demonstrated in the four panels.** The horizontal dashed line **(A,C,E,G)** indicates the genome-wide significance threshold (*P* = 3.8 × 10^7^).

Six heading date QTLs in *indica* subpopulation and no QTLs in *japonica* subpopulation were detected under SD (**Figure 3**; **Table 4**). Two QTLs (*qHd6.3* and *qHd7.1*) on chromosomes 6 and 7 were repeatedly detected in 2 years *indica* subpopulation. *qHd6.3* and *qHd7.1* primarily explained 26.5% and 10.0% of the phenotypic variations, respectively. In total, the QTLs explained 57.7% and 41.3% of the phenotypic variation, respectively, in 2 years in *indica* subpopulation. Three QTLs were located near known heading date genes: the lead SNP Sf709158944 for *qHd7.1* was 6.5 kb to *Ghd7*, the lead SNP Sf1017100637 for *qHd10.1* was 24.5 kb to *Ehd1* and the lead SNP Sf1102480009 for *qHd11* was 27.5 kb to *RCN1.*

**Table 4:** Genome-wide significant association signals for heading date in the *indica* subpopulations using the LMM method in Hainan (short day conditions).

QTL	Lead SNP	Chr.	Linkage disequilibrium interval (bp)	Distance to gene	*Indica* 2012		*Indica* 2013	
					P	P.V (%)	P	P.V (%)
*qHd3*	Sf0322143338	3	22079360–22143338		1.4E-8	23.3		
*qHd6.3*	Sf0607922178	6	7917158–8424734		1.3E-9	22.0	1.1E-8	26.5
*qHd7.1*	Sf0709158944	7	9137593–9196135	-6.5 kb to *Ghd7*	1.2E-7	7.7	2.0E-6	10.0
*qHd7.3*	Sf0712842846	7	12774834–12894276		1.4E-7	1.1		
*qHd10.2*	Sf1017100637	10	16691541–17385511	-24.5 kb to *Ehd1*			2.9E-7	4.7
*qHd11*	Sf1102480009	11	2357177–2488304	-27.5 kb to *RCN1*	3.8E-07	3.5		

Total estimation					2.1E-46	57.7	1.0E-30	41.3

*Linkage disequilibrium interval means the linkage disequilibrium (r^2^> 0.2) extended genome region surrounding the lead SNP. P is P-value estimated in LMM, P.V (%) is phenotype variance explained by the QTL in percent.*

### Haplotype-Level Association Analysis of 11 Known Heading Date Genes

To date, dozens of heading date genes have been cloned in rice. However, only four QTLs in our study were located near heading date genes, which were less than 80 kb from the corresponding lead SNPs. The average linkage disequilibrium decay in these *indica* and *japonica* groups extended 93 and 171 kb, respectively^4^, which was similar to the estimates of 75–123 kb in *indica* and 150–167 kb in *japonica* in other studies (Zhao et al., 2011; Huang et al., 2012). In our study, lead SNPs pointed to the four QTLs (*qHd6.1*, *qHd7.1*, *qHd10.1*, and *qHd11*) were in linkage disequilibrium with known heading date genes of *Hd17*, *Ghd7*, *Ehd1*, and *RCN1*, respectively (**Tables 3** and **4**). Thus, these heading date genes most likely underlined these four QTLs. We questioned whether known heading gene had a greater chance to be associated by haplotype, a combination of a set of SNPs within gene. We then extracted SNPs from the initiation codon to the termination codon of 11 genes: *Hd17*, *Ghd7*, *Ehd1*, *RCN1*, *Hd1*, *Ghd8*, *Ghd7.1*, *DTH2*, *Hd3a*, *OsMADS50*, and *OsMADS56*; haplotypes were then constructed. Most of these genes had more haplotypes in *indica* subpopulation than *japonica* subpopulation, with the exception for *Ghd7.1* and *OsMADS56* (**Table 5**). The associations were re-estimated at the haplotype level. The four heading date genes detected using GWAS at the SNP level were identified via haplotype association. Accordingly, *Ghd7* was identified in the *indica* subpopulation under both SD and LD. However, *RCN1* was detected in *indica* under SD, and *Hd17* was detected in both subpopulations under LD. *Ehd1* was detected in *japonica* under SD. Additionally, *Ghd7.1*, *Hd3a*, and *OsMADS56* were detected via haplotype association (**Supplementary Figure S1**; **Table 5**). *Ghd7.1* was identified in *japonica* subpopulation under both SD and LD. *OsMADS56* was specifically identified under SD in *japonica* subpopulation. However, *Hd3a* was specifically identified in *indica* under LD.

**Table 5:** Association analyses for known heading date genes in the *indica* and *japonica* subpopulations based on haplotypes using 425 accessions.

Gene	N (Ind, Jap)^∗^	Subpop	Wuhan		Hainan	
			P (2013)	P (2014)	P (2012)	P (2013)
*Hd17*	6 (6, 2)	Ind	9.4E-06	2.6E-08		
		Jap	1.3E-08	1.5E-08		
*Hd3a*	6 (6, 2)	Ind	1.8E-07	3.8E-09		
*RCN1*	3 (3, 1)	Ind			2.6E-11	2.3E-08
*Ehd1*	5 (3, 3)	Jap			5.3E-16	2.0E-12
*OsMADS56*	7 (3, 4)	Jap			1.8E-09	6.3E-09
*Ghd7*	6 (4, 2)	Ind	1.5E-19	8.2E-17	3.1E-18	9.4E-10
*Ghd7.1*	14 (5, 10)	Jap	1.5E-11	5.9E-11	3.5E-21	1.9E-23

*^∗^N was the number of haplotypes in 425 accessions. The numbers inside the parentheses are the numbers of haplotypes in the indica and japonica subpopulations, respectively. P is P-value in ANOVA.*

### Haplotype Analysis for *Ghd7* and *OsMADS56*

*Ghd7* is a key regulator of heading date and has five haplotypes with strong, weak or null-functions in cultivars (Xue et al., 2008; Lu et al., 2012). To obtain insight into why *Ghd7* was not detected in *japonica*, we compared the haplotypes of *Ghd7* in *indica* and *japonica* subpopulations (**Table 6**). In addition to the previously reported five haplotypes, one additional haplotype (*Ghd7*-4) was identified. In *indica* subpopulation, strong alleles of *Ghd7*-1 and *Ghd7*-3, weak allele of *Ghd7*-4 and non-functional allele of *Ghd7*-0 were observed, which caused a large variation in heading date and resulted in a successful detection. However, in *japonica* subpopulation, we only detected weak functional alleles of *Ghd7*-2 and non-functional alleles of *Ghd7*-0a, resulted a small variation and failed detection. The similar result was observed for *OsMADS56* (**Table 7**). That is, *indica* rice carried haplotypes with similar genetic effects on heading date and *japonica* rice carried haplotypes with distinct genetic effects, these data explain why *OsMADS56* was detected in only *japonica* subpopulation.

**Table 6:** Comparison of heading date (days) among haplotypes of *Ghd7* in *indica* and *japonica* subpopulations.

Pop	Haplotypes	Number	Hainan 2012	Hainan 2013	Wuhan 2013	Wuhan 2014
*Indica*	*Ghd7*-1	161	98.7 ± 11.4C	98.4 ± 7.7C	101.4 ± 11.5C	103.7 ± 11.7C
	*Ghd7*-3	70	96.0 ± 10.1C	96.6 ± 7.0C	96.6 ± 6.8B	97.4 ± 6.0B
	*Ghd7*-4	18	72.4 ± 12.4B	86.4 ± 7.9B	76.8 ± 10.9A	82.5 ± 12.4A
	*Ghd7*-0	6	79.8 ± 16.6B	88.1 ± 10.1B	78.7 ± 11.2A	82.6 ± 9.1A
*Japonica*	*Ghd7*-2	136	87.2 ± 22.3b	94.4 ± 12.2b	97.3 ± 17.2b	100.0 ± 17.4b
	*Ghd7*-0a	5	59.0 ± 5.1a	79.9 ± 4.2a	61.0 ± 5.7a	68.2 ± 4.5a

*The phenotype values are represented as the means ± SD, letters are ranked via Duncan test at P < 0.01.*

**Table 7:** Comparison of heading date among haplotypes of *OsMADS56* in *indica* and *japonica* subpopulations in Hainan.

Pop	Haplotypes	Number	Hainan 2012	Hainan 2013
*Indica*	*OsMADS56*-1	59	97.5 ± 10.9A	98.0 ± 7.8A
	*OsMADS56*-2	145	93.3 ± 15.2A	95.7 ± 9.0A
	*OsMADS56*-3	28	99.8 ± 11.1A	99 ± 7.9A
*Japonica*	*OsMADS56*-4	64	73.7 ± 18.5a	87.9 ± 7.6a
	*OsMADS56*-5	13	101.5 ± 20c	101.5 ± 10.5c
	*OsMADS56*-6	32	92.7 ± 22.8b	95.9 ± 13.5b
	*OsMADS56*-7	9	111.6 ± 8.1d	108.4 ± 7.0d

*The phenotype values are represented as the means ± SD, letters are ranked via Duncan test at P < 0.01.*

## Discussion

### Diverse Genetic Basis of Heading Date between *Indica* and *Japonica* Subpopulations

The large range in heading dates in the *indica* subpopulation was similar to that in the *japonica* subpopulation in each environment (**Figure 1**), which indicated a complicated genetic basis heading date in rice. However, no common QTLs were detected at the SNP level in two subpopulations (**Tables 3** and **4**). Under LD, *Hd17* and *Ghd7* were the primary heading genes in *indica* subpopulation; whereas *qHd4*, *qHd6.2*, and *qHd7.2* were the primary players in *japonica* subpopulation. Under SD conditions, there were no genomic regions associated with heading date in *japonica* subpopulation, which indicated that the heading date genes in *japonica* had a photoperiod sensitivity that was too weak to efficiently function under SD conditions and led to a failed detection (**Table 4**). On contrary, a few QTLs were identified in *indica* subpopulation, which indicated that *indica* rice carried strong photoperiod-sensitive genes, which affected heading date even under SD. These results show that *indica* and *japonica* subpopulations have different gene/allele responses to day length.

The power of association mapping was greatly improved at the haplotype level; however, only one heading gene (*Hd17*) was commonly identified in both *japonica* and *indica* subpopulations under LD (**Table 5**). Moreover, the 7 associated known heading genes (with the exception of *OsMADS56* and *Ghd7.1*) had more haplotypes in *indica* than *japonica*, which indicated that *indica* rice in regulating heading date was more genetically diverse than *japonica*. This result is in agreement with the previous finding that the genetic diversity in *indica* (0.0016) was much higher than that (0.0006) in *japonica* (Huang et al., 2010). There were 10 alleles of *Ghd7.1* in *japonica* and five alleles in *indica*; however, only one allele was common across the two subpopulations. This finding was consistent with the reported assumption that *indica* and *japonica* alleles of *Ghd7.1* evolved independently (Zhang et al., 2013). In most cases, *japonica* rice carries weak or non-functional alleles of photoperiod-sensitive genes, which enable it to be grown at high latitudes with LD during the rice-growing season. *Indica* rice carries diverse alleles, including strong, weak and non-functional alleles, which enable its growth across various climatic regions.

### Different Heading Date Gene Responses to Long and Short Day Lengths

In this study, we detected QTLs for heading date using GWAS under SD (Hainan) and LD (Wuhan). At the SNP level, no common QTL was identified between SD and LD in *japonica* (**Tables 3** and **4**). In *indica*, only the known major gene *Ghd7* was commonly detected under both conditions. For the 11 known heading date genes, seven were identified at the haplotype level. However, only *Ghd7* in *indica* and *Ghd7.1* in *japonica* were commonly detected under both conditions (**Table 5**). This indicated that *Ghd7* and *Ghd7.1* simultaneously contributed to the variation in heading date under both conditions, which is in agreement with the findings that *Ghd7* and *Ghd7.1* primarily function under LD, and they have weak effects under natural short day conditions (Xue et al., 2008; Zhang et al., 2013). As we expected, *Ehd1*, a major heading date gene that primarily functions under SD conditions (Doi et al., 2004), was detected under SD conditions. Accordingly, the gene *Hd17*, which primarily functions under LD conditions (Matsubara et al., 2012), was detected under LD conditions. *Hd3a*, a rice florigen gene with expression that is repressed under LD and promoted under SD (Tamaki et al., 2007; Tsuji et al., 2011), was detected under LD in this study. This finding indicated that probably the upstream regulators act with *Hd3a* more actively under LD than SD. However, *OsMADS56* and *RCN1*, two major heading date genes primarily working under LD (Nakagawa et al., 2002; Ryu et al., 2009), were detected under SD conditions. This inconsistency indicated that these genes likely have a new function, or association mapping is not sufficiently powerful so that some key genes are not detected. These unknown QTL detected under SD or LD may primarily regulate heading date dependent on short or long day length, respectively. Different QTL detected under SD and LD suggested that heading date genes have various photoperiod sensitivities. A parallel experimental design similar to this study is suggested for identifying and characterizing the genes with responses to environmental factors such as temperature or fertilizers. No heading date genes have been reported around the major QTL *qHd3* in *indica* under SD and *qHd4* in *japonica* under LD. They are likely new heading date genes. Populations designed for mapping both QTL are encouraged to confirm their identities.

### Haplotype-Level Association Analysis Improved the Power of Mapping

In rice, several papers reported GWAS for important agronomic or biological traits at the SNP level. However, at least some or even a large portion of known functional genes was not identified (Zhao et al., 2011; Huang et al., 2012). The most likely reason for this is that in the most cases, SNP classification divided the collection into only two classes, whereas a gene has multiple alleles exhibiting different genetic effects in a natural population. Therefore, an SNP-derived low-resolution classification confused functional and non-functional alleles that were distinguished in multiple SNP combinations and compromised the real differences between alleles, thereby producing a failed detection. A haplotype is the genetic constitution of one locus/gene, which is specified by a minimum number of SNPs. At the single gene level, a haplotype is equal to an allele. In consequence, a haplotype-level GWAS is expected to mine more genes hidden in the whole genome because a haplotype-level GWAS shows the difference between haplotypes rather than conflating these differences.

Notably, most genes excluding housekeeping genes include several haplotypes/alleles, such as *Ghd7* and *OsMADS56*, in the germplasm collection (**Tables 6** and **7**). Only four known heading genes (*Ehd1*, *Hd17*, *RCN1*, and *Ghd7*) were identified at the SNP level. However, for the 11 known genes, seven (including the four detected via SNP-level GWAS (SGWAS)) were identified via haplotype-level association analysis (**Supplementary Figure S1**; **Table 5**). Moreover, the order of magnitudes of the *P*-values for these four genes was greatly decreased comparing with SGWAS (**Tables 3**–**5**). Obviously, haplotype-level association analysis immensely increased the mapping power. In this study, we choose only 11 known genes as candidates to test the haplotype-level association analysis. Moreover, haplotype analysis ranked the genetic effects of haplotypes, which provided favorable haplotypes for breeding design. However, a haplotype-level genome-wide association study (HGWAS) faces substantial challenges because several points should be considered when constructing genome-wide haplotypes. (i) The haplotype specification should be made per gene unit or coding sequence of one gene that potentially matches a phenotypic variation, (ii) The species has a high-quality reference genome sequence for SNP screening and high-quality full-length cDNA sequences to delimit the gene region, (iii) The minimum deep sequencing and high-quality sequencing data are required for the identification of haplotypes by decreasing imputations of the missing and heterozygous sites and alleviating the calculation burden. The association mapping methods should also be further developed for HGWAS.

## Conclusion

GWAS revealed that diverse genes/QTLs between *indica* and *japonica* subpopulations regulated heading date under SD and LD. Test of haplotype-level association mapping for 11 known heading date genes confirmed that the power of association mapping was greatly improved as compared to SNP-level association mapping and HGWAS approach is encouraged to develop in rice.

## Author Contributions

ZH performed the experiments, analyzed the data and wrote the paper. BZ performed the experiments, HZ analyzed data, and MA wrote the paper. YX conceived the project, designed the research, and wrote the paper.

## Conflict of Interest Statement

The authors declare that the research was conducted in the absence of any commercial or financial relationships that could be construed as a potential conflict of interest.

## Footnotes

1 http://ricevarmap.ncpgr.cn

2 http://ricevarmap.ncpgr.cn

3 http://www.r-project.org/

4 http://ricevarmap.ncpgr.cn

## Supplementary Material

FIGURE S1 **Physic map of associated QTLs and candidate genes via SNP-level GWAS (SGWAS) and haplotype-level GWAS (HGWAS)**. The black QTLs are detected by SGWAS. The red heading date genes are detected by HGWAS. The blue heading date genes are detected by both SGWAS and HGWAS. The purples on the left side of each chromosome are heading date genes are failed in detection by GWAS.
