# Supplementary figures and images for: Genome-Wide Association Studies Reveal that Diverse Heading Date Genes Respond to Short and Long Day Lengths between Indica and Japonica Rice
Source: Front Plant Sci. 2016 Aug 29;7:1270. doi: 10.3389/fpls.2016.01270 (PMC5002401; doi:10.3389/fpls.2016.01270)

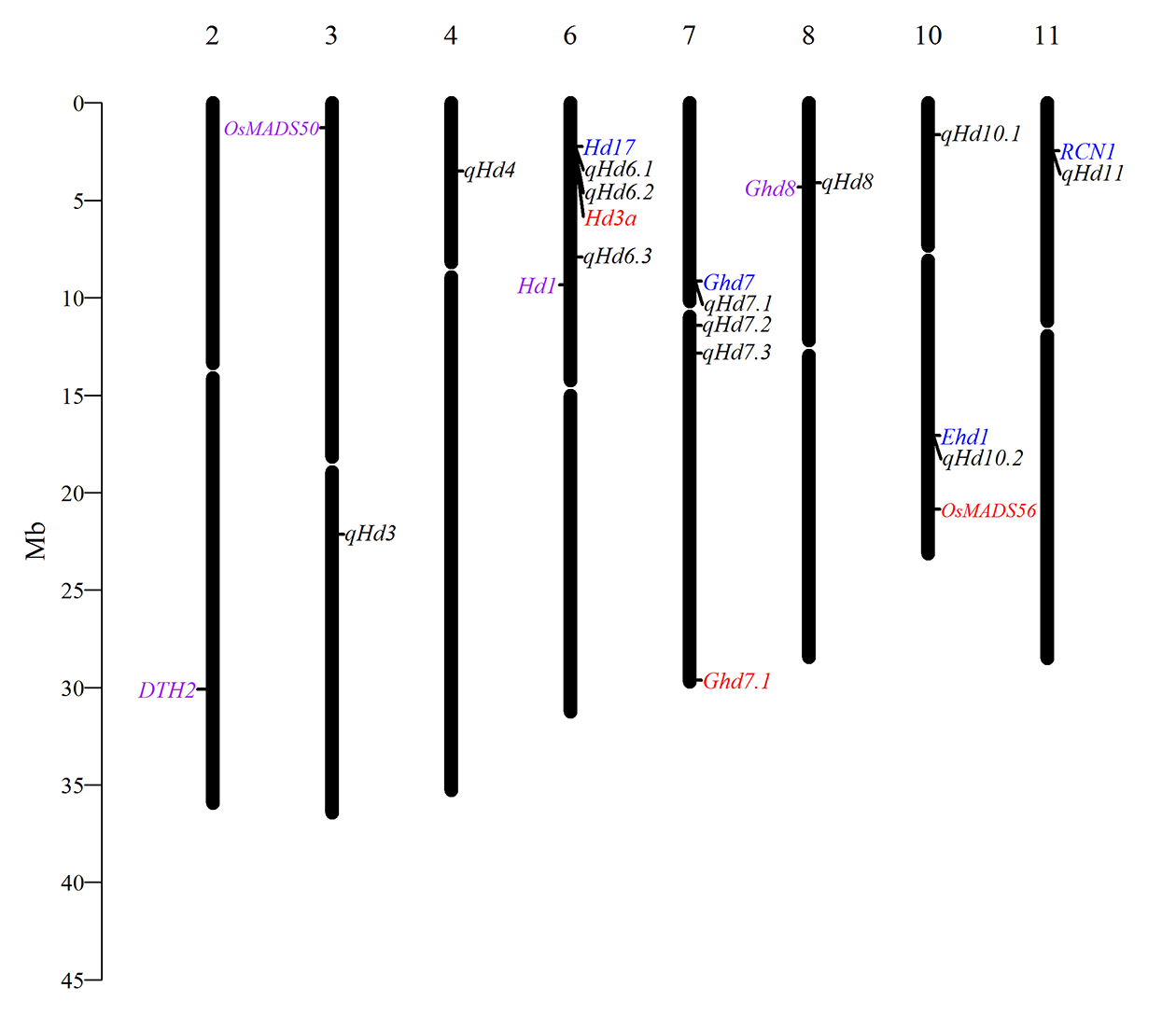

Supplement: FIGURE S1 — Physic map of associated QTLs and candidate genes via SNP-level GWAS (SGWAS) and haplotype-level GWAS (HGWAS). The black QTLs are detected by SGWAS. The red heading date genes are detected by HGWAS. The blue heading date genes are detected by both SGWAS and HGWAS. The purples on the left side of each chromosome are heading date genes are failed in detection by GWAS. [file Image_1.TIFF]
